# GraphGPT: A Graph Enhanced Generative Pretrained Transformer for Conditioned Molecular Generation

**DOI:** 10.3390/ijms242316761

**Published:** 2023-11-25

**Authors:** Hao Lu, Zhiqiang Wei, Xuze Wang, Kun Zhang, Hao Liu

**Affiliations:** College of Computer Science and Technology, Ocean University of China, Qingdao 266100, China

**Keywords:** molecular generation, generative pretrained transformer, graph neural networks

## Abstract

Condition-based molecular generation can generate a large number of molecules with particular properties, expanding the virtual drug screening library, and accelerating the process of drug discovery. In this study, we combined a molecular graph structure and sequential representations using a generative pretrained transformer (GPT) architecture for generating molecules conditionally. The incorporation of graph structure information facilitated a better comprehension of molecular topological features, and the augmentation of a sequential contextual understanding of GPT architecture facilitated molecular generation. The experiments indicate that our model efficiently produces molecules with the desired properties, with valid and unique metrics that are close to 100%. Faced with the typical task of generating molecules based on a scaffold in drug discovery, our model is able to preserve scaffold information and generate molecules with low similarity and specified properties.

## 1. Introduction

Drug discovery and development constitute a complex and challenging process demanding extensive time, resources, and domain expertise [1,2,3,4]. To address this issue, virtual screening methods have emerged as pivotal tools for efficiently identifying potential drug candidates [5,6,7]. Currently, the virtual screening library can be scaled up to the billion level and is gradually increasing [8,9,10]. However, since the lead compound does not meet certain essential drug properties, including topological polar surface area, lipophilicity, etc., subsequent molecular optimization is required, which may even lead to drug development failures. Therefore, condition-based molecular generation can not only build high-quality molecular virtual screening libraries but also plays an important role in lead compound optimization, which in turn, drives drug development.

Molecular generation models based on artificial intelligence (AI) have showcased immense potential and have become pivotal in de novo drug design [11,12]. These models encompass the Variational Autoencoder (VAE) [13], the Generative Adversarial Network (GAN) [14], and Reinforcement Learning (RL) [15]. The reward function of RL can quickly focus on a chemical region while causing instability in the model [16,17], e.g., LS-MolGen [18] and DrugEx v3 [19]. Transfer learning mitigates this problem but results in generated molecules that are similar to the molecules in the training set due to the rapid concentration on a certain target region [20]. Generative pretrained language models have exhibited tremendous potential across various domains [21,22,23]. Molecular generation methods based on language models have demonstrated impressive capabilities in SMILES [24] syntactic representation [25,26]. These methods grapple with a significant challenge: the inability to capture molecular topological information. Conventional sequence-to-sequence models often disregard the intrinsic spatial arrangement of atoms, resulting in an incomplete representation of molecular topology [27]. Transformer [28] and graph neural networks are currently combined in works like GraphTransformer [29] and the Structure-Aware Transformer (SAT) [30]. Nevertheless, unlike supervised learning methods, molecular generation models lack explicit structural information during the generation process [31]. Consequently, these models are unable to fully incorporate the structural features of molecules into the generation process, thereby limiting their capacity to generate molecules with specific topological properties.

**Figure 1:**
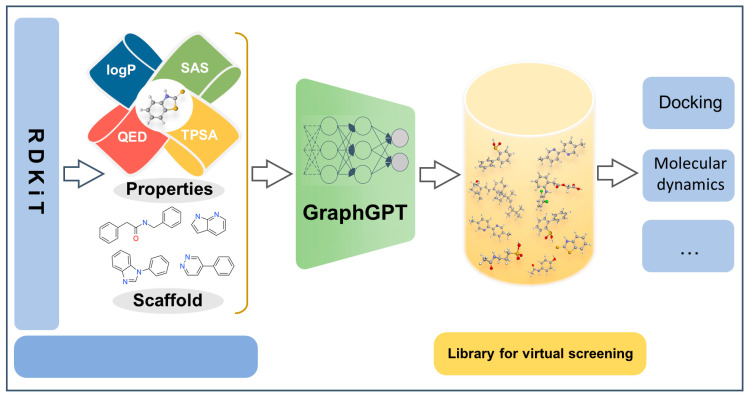
Condition-based molecular generation and downstream applications of virtual screening library.

In this work, we focus on the molecular generation task to produce molecules with designated properties, thereby expanding molecular screening libraries (Figure 1). We proposed a graph neural network augmented GPT model called GraphGPT. To compensate for the lack of topology of molecules characterized by SMILES, we introduce an innovative methodology that integrates graph-based representations into the sequence-to-sequence paradigm. This innovative approach enables our model to maintain and leverage crucial structural information during the molecular generation process. By amalgamating the advantages of graph-based representation and language modeling, our approach provides a more comprehensive and contextually enriched avenue for molecular generation.

## 2. Results

### 2.1. Molecular Generation Based on Properties

To test the ability of our model to generate molecules with a specific property, we specified a single property (Synthetic Accessibility Score (SAS)) [32], Quantitative Estimation of Drug-likeness (QED) [33], lipophilicity (logP) [34], and the Topological Polar Surface Area (TPSA) [35]) of the molecule to generate the molecule separately. We evaluated the performance of molecular generation using metrics that include validity, uniqueness, and novelty.

As shown in Table 1, we observed that both the MolGPT [36] and our model exhibit a relatively similar performance across the four properties. These metrics are close to one in terms of being valid and unique, indicating the capability of both models to generate high-quality molecular structures, with the majority of these molecules being unique. Furthermore, both models achieve a novelty value of one, indicating that the generated molecules are not present in the training dataset, thus mitigating overfitting concerns. Attention should also be paid to the standard deviation (SD) and mean absolute deviation (MAD) metrics. These metrics gauge the stability and consistency of the results. A smaller standard deviation and mean absolute deviation signify a more stable and consistent distribution of samples. It is evident that our model exhibits smaller standard deviations and mean absolute deviations in most SD/MAD metrics, indicating a greater stability compared to the MolGPT. This suggests that our model yields more consistent results across multiple experiments, displaying minimal performance fluctuations.

**Figure 2:**
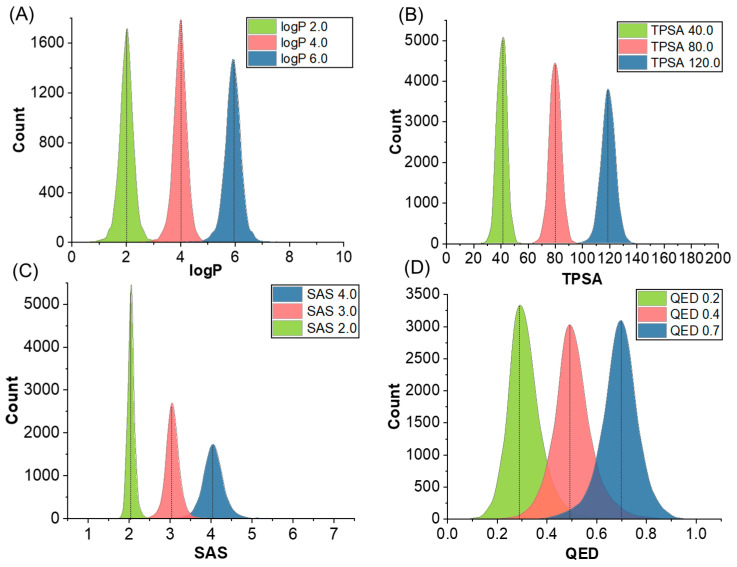
Generating property distribution from single-property molecules based on the model trained on the GuacaMol dataset; molecular properties include logP (**A**), TPSA (**B**), SAS (**C**), and QED (**D**). The legend in the upper right-hand corner represents the combinations of molecular properties that we defined.

In the context of generating a single property, our findings demonstrate (Figure 2) that the generated molecules exhibit desirable properties in terms of logP, SAS, and TPSA, affirming that the model effectively generates molecules with specific properties. It is noteworthy that some deviations were observed in the QED property, indicating a need for further refinement to ensure precise alignment with this metric. QED describes the drug-likeness of molecules, influenced by multiple underlying properties. As such, the model might face challenges in accurately controlling QED. These observations underscore the capability of our model in tailoring molecular properties while also highlighting the necessity to enhance its performance in generating molecules with specified QED.

**Figure 3:**
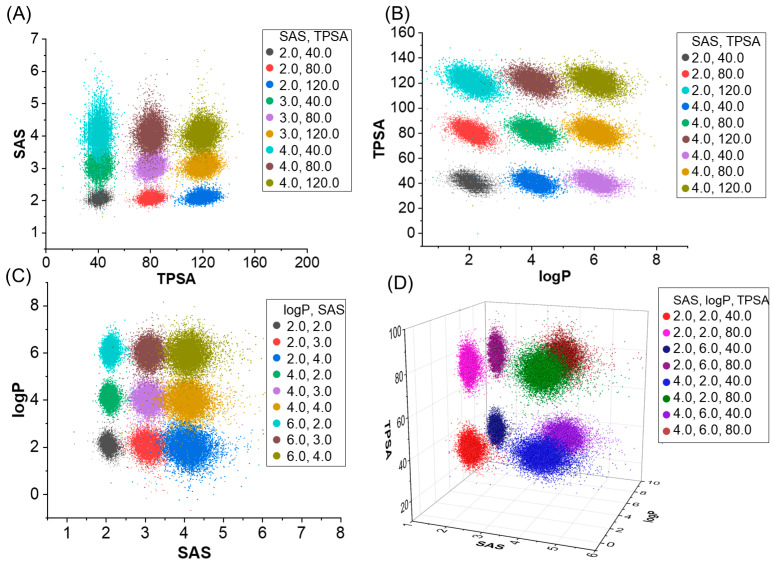
Distribution of molecular properties generated when specifying multiple properties using a model trained on the GuacaMol dataset. SAS and TPSA (**A**), logP and TPSA (**B**), SAS and logP (**C**), SAS, logP and TPSA (**D**). The legend in the upper right-hand corner represents the combinations of molecular properties that we defined.

Concurrently, we tested the performance of molecular generation based on multi properties. Table 2 and Figure 3 contain the evaluation results of two models, involving property and evaluation metrics such as validity, uniqueness, and novelty, and MAD/SD as a stability metric of the results. First of all, we can see that MolGPT and our model perform similarly under most combinations of properties, with scores very close to one for validity, uniqueness, and novelty, indicating that they are both capable of generating high-quality, unique, and novel molecular structures. MAD and SD are indicators used to assess the stability of molecular samples generated by our model. A smaller MAD and SD imply a more stable and consistent distribution of results. As can be seen from Table 2, the MAD and SD of our model are generally smaller for all combinations of properties. Our model is able to establish the relationship between molecular structure and molecular properties by integrating topological information, thereby demonstrating improved multi-property generation.

In summary, although MolGPT and our model perform similarly in terms of validity, uniqueness, and novelty, our model is superior in terms of the stability of the results, i.e., the generated samples are more consistent and reliable across multiple experiments. It can be concluded that our model is better in this molecular generation task, and the generated molecular structures are not only of high quality but also show more stable performance in different specified properties experiments.

### 2.2. Molecular Generation Based on Properties and Scaffold

The molecular scaffold constitutes a pivotal element in drug design, influencing the structure, properties, and interactions of molecules. By strategically designing and modifying the molecular scaffold, drug molecules can attain specific biological activities, pharmacokinetics, and safety profiles, thereby laying a robust foundation for novel drug development. Therefore, we tested the performance of GraphGPT in generating molecules for a given scaffold and properties.

**Figure 4:**
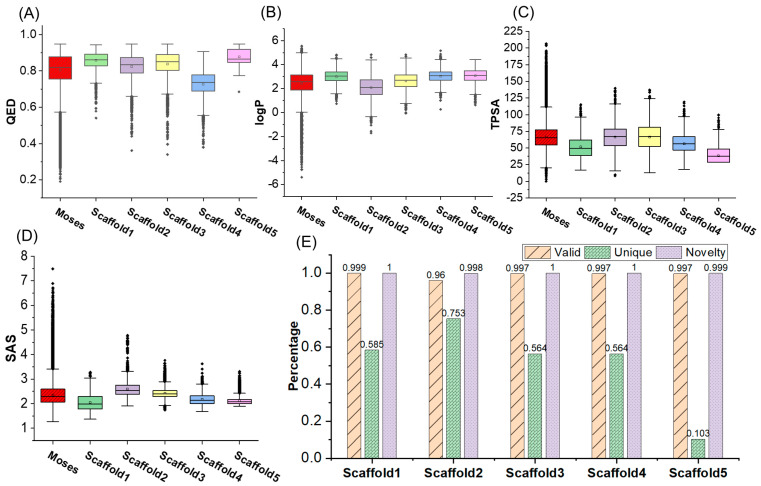
Properties of generated molecules based on five scaffolds and a single property. (**A**–**D**) are box-and-line plots for generated molecules with specified QED, logP, TPAS, and SAS, respectively. (**E**) is validity, uniqueness, and novelty of the generated molecules.

We adopted the five molecular scaffolds used by Bagal et al. [36], and tested the molecular generation based on these molecular scaffolds and single property. A boxplot was constructed by calculating the QED, logP, TPSA, and SAS of generated molecules, as illustrated in Figure 4A–D. Except for the presence of several outliers in the QED property, the molecular properties of the molecules generated based on the five molecular scaffolds were mostly within 1.5 of the interquartile range (IQR). Moreover, the means and medians of the various properties of the generated molecules were similar to those in the Moses dataset. Furthermore, Figure 4E indicates that the novelty of the molecules generated from the five molecular scaffolds exceeded 0.998, with valid samples of approximately 0.96. As shown in Section 2.4, the atom sizes of the molecular scaffold we used vary, with scaffold5 having up to 19 heavy atoms and scaffold2 having 9 heavy atoms. The uniqueness of the generated molecules based on both scaffold and single property exceeds 0.5, except for scaffold5. The unique metric was above 0.564 for the four sets of generated molecules, whereas scaffold5 exhibited a uniqueness value of 0.103. This difference of 0.65 compared to the corresponding indicator for scaffold2 might be attributed to the larger molecular size of scaffold5, resulting in a smaller space for the generated molecules.

**Figure 5:**
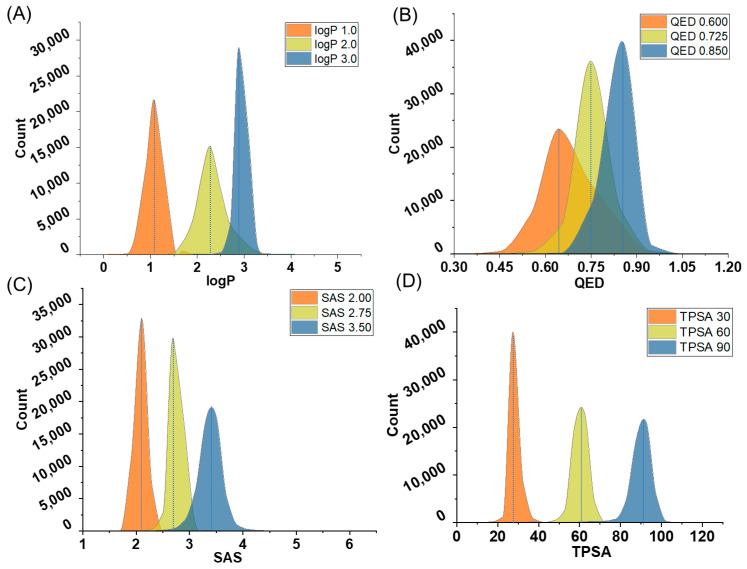
Distribution of molecular properties for molecular generation based on five molecular scaffolds and a single property, (**A**–**D**) are distribution of generated molecules specifying logP, QED, SAS and TPSA, respectively. The legend in the upper right-hand corner represents the combinations of molecular properties that we defined.

**Figure 6:**
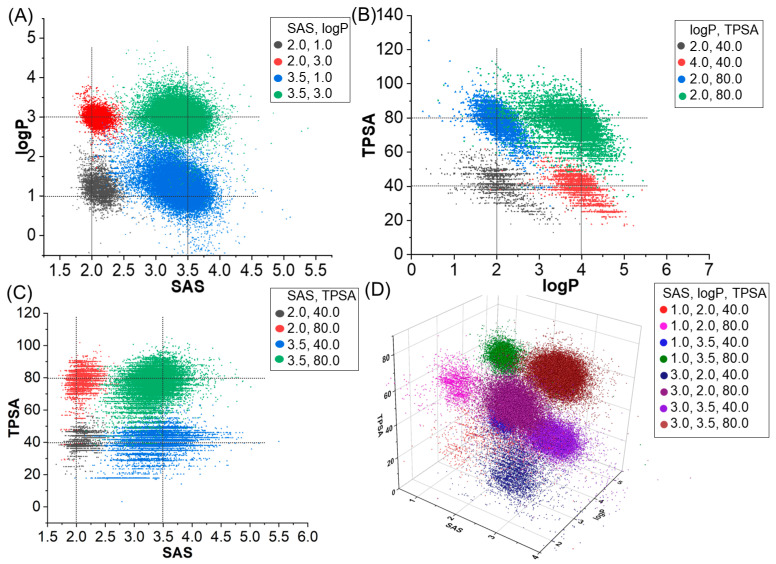
Molecular generation samples of multiple properties and scaffold. (**A**) SAS, LogP and scaffold. (**B**) LogP, TPSA, and scaffold. (**C**) SAS, TPSA, and scaffold. (**D**) SAS, LogP, TPSA, and scaffold. The legend in the upper right-hand corner represents the combinations of molecular properties that we defined.

We visualize the properties of the generated samples in the scaffold and single property-based molecular generation experiments. As can be seen in Figure 5, the model is largely able to generate molecules according to a specified scaffold and a single property. Similar to the single property generation experiment without a scaffold, the molecules generated with the specified QED are more dispersed. In the case of specifying the scaffold and three properties (Figure 6), the quality of molecule generation is poorer under SAS: 1.0, logP: 2.0, and TPSA: 40.0, which may be due to the lower property coverage of these properties for the training set molecules. The molecule generation results were better in other cases.

### 2.3. Unconditional Molecular Generation

We conducted unconditional molecular generation on the GuacaMol [37] dataset, sampling 10,000 molecules and calculating various metrics for the generated molecules. The FCD [38] metric of the molecules significantly exceeded that of other models, reaching 1.009 (Table 3). Additionally, the KL divergence [39] metric matched that of the MolGPT model at 0.992, suggesting that it had a strong grasp on the training data distribution. While the validity of the generated molecules experienced a slight decrease, both uniqueness and novelty levels remained high. This indicates that the model, while capable of generating high-quality molecules, also learned the statistical characteristics of the trained molecules.

As shown in Table 4, when trained on datasets like the Moses dataset [40] containing drug-like small molecules, the model demonstrated a slight improvement in the validity metric while experiencing marginal reductions in uniqueness and novelty. The slight decrease in novelty is attributed to the model’s improved ability to learn a more accurate representation of the molecules in the dataset, as a result of incorporating topological information. This leads to the generation of molecules that more closely resemble those in the training set. The IntDiv1 and IntDiv2 metrics for molecule diversity saw an increase. This demonstrates that our model can generate high-quality molecules in an unconstrained scenario.

### 2.4. Case Study

**Figure 7:**
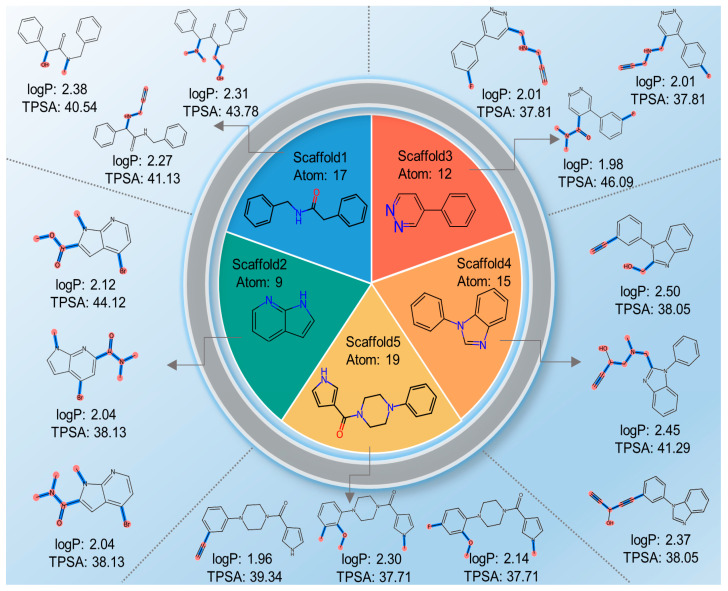
Samples of the generated molecules based on the five molecular scaffolds and properties (logP: 2, TPSA: 40.0). The generated bonds are represented in blue, and the atoms in light red.

We employed the model trained on the Moses dataset for molecular generation, predefining the molecular scaffold, logP, and TPSA before generating molecules. Five molecular scaffolds were used to test the performance of molecular optimization. LogP was set to 2.0, and TPSA was set to 40.0. In other words, we aimed to generate molecules that retained their molecular scaffolds while achieving logP and TPSA values close to 2.0 and 40.0, respectively. Partially sampled molecules are illustrated in Figure 7; it can be observed that the generated molecules preserved the specified molecular scaffold while approximating the predetermined properties. Hence, our model can achieve molecular generation based on both molecular scaffold and properties.

### 2.5. Ablation Experiment

To verify the effect of different decoder layers as well as graph encoders on miniGPT, we performed ablation experiments. As shown in Table 5, three values of 40, 80, and 120 are set in the specified TPSA, and it can be seen that there is little difference in the validity, uniqueness, and novelty metrics. As the number of layers in the decoder increases, the standard deviation and MAD gradually become smaller, indicating the model’s ability to simulate the properties of SMILES (the top three rows of Table 5). Considering that as the number of decoder layers increases the molecular validity, uniqueness, and novelty are close to one, we added graph encoders to the model, with eight decoders for runtime as well as efficiency reasons. The addition of the graph encoder reduces SD by 0.266 and MAD by 0.178 under the condition that all eight decoders are used (miniGPT_b and GraphGPT). The validity of the molecule is reduced by 0.001, which is acceptable for the sake of coincidence. After adding the graph encoder, the standard deviation of the numerator is even smaller, proving the effectiveness of the graph encoder.

### 2.6. Attention Visualization

**Figure 8:**
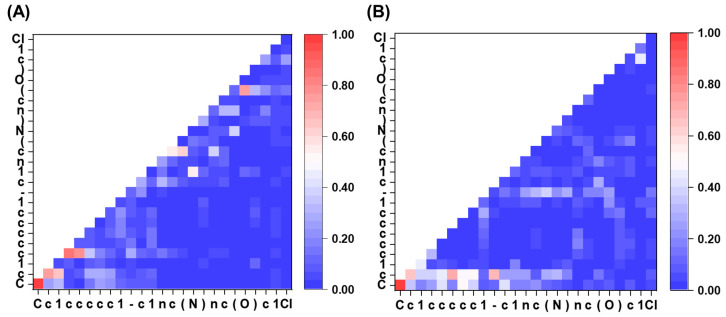
Attention heatmap between tokens in the last decoder layer. (**A**) Using the sequence encoder only, and (**B**) using GraphGPT.

Figure 8 depicts an attention heatmap between tokens in the final layer of the model before and after encoding with a graph. The attention heatmap offers a visual interpretation of the representation of SMILES by the model. It is evident that, during the characterization of the molecule using GraphGPT, all tokens in the first part of the structure (“Cc1ccccc1”) are strongly focused on the first “c”, except for “C”. This results in the formation of toluene and the presence of a significant number of benzene ring structures in the drug, which is consistent with chemical knowledge. Furthermore, the model places greater emphasis on the non-atomic tokens such as “1” and “-”. This indicates the significance of accessory tokens in SMILES for the process of molecular generation. This behavior may be due to the model searching for signs of the conclusion of a functional group or other factors.

## 3. Discussion

We use a GAT-based graph encoder to encode the molecular structure in order to solve the problem of missing topology in the sequence-based molecular generation process. The alignment of the graph encoder with the sequence encoder enables the fusion of structural information into the sequence encoder. Experiments demonstrate that the model is able to generate more accurate molecular properties with little degradation in performance such as molecular validity. There are also variants of SMILES such as SELFIES [41], R-SMILES [42], etc., which encode molecules, and in the future, we can try to use this sequence information for molecule generation. In addition, we did not try to take information such as target activity into account, which is the main area for our future research.

## 4. Methods and Materials

In this section, the overarching architecture of the model is initially presented. Subsequently, the structure of the graph-based encoder is expounded upon. Following this, the encoding of molecular scaffolds, properties, and molecular SMILES sequences are delineated through the utilization of a sequence encoder akin to the GPT framework. Lastly, the employed loss function is elucidated, facilitating the realization of molecular sequence generation fortified by graph-enhanced structures.

### 4.1. Overview of the Model

**Figure 9:**
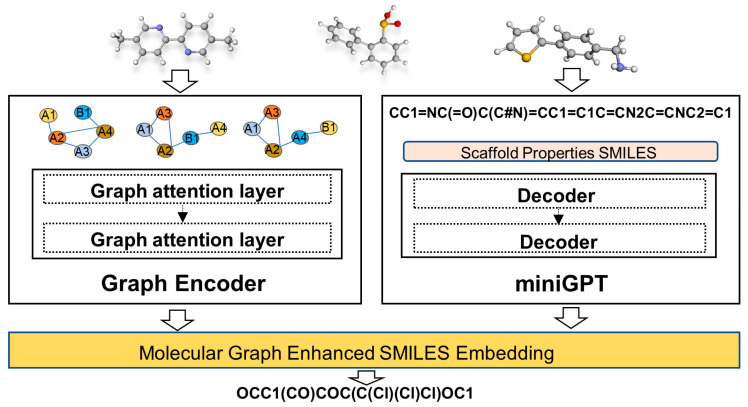
Architecture of GraphGPT.

A sequence-to-sequence molecular generation model enhanced with graph structures was proposed. This model was capable of generating molecules based on molecular scaffolds and properties. As illustrated in Figure 9, the approach commenced by employing a graph-based encoder to encode the molecular structures, with the aim of capturing the inherent graph-related information of the molecules and thereby creating molecular encoding inclusive of graph-based structures. Subsequently, employing techniques from natural language processing, the molecules were represented using SMILES, facilitating the extraction of relationships between molecular properties and sequences, as well as capturing the syntactical format of SMILES. Lastly, a loss function was formulated and devised to seamlessly integrate the structure of molecules within the sequential data, thereby accomplishing the amalgamation of structural insights into the sequence generation process.

### 4.2. Graph Encoder

**Figure 10:**
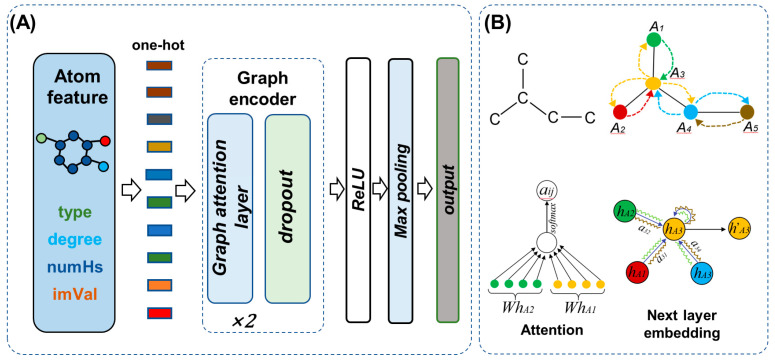
Details of graph encoder. (**A**) Graph encoding of molecules, type, degree, NumHs, and imVal refer to atom type, degree, amount of hydrogen, and implicit valence, respectively. (**B**) Graph attention mechanism.

To address the deficiency in capturing the previous molecular topology within the molecular generation model, we introduced the Graph Attention Mechanism (GAT) [43] for encoding the structural aspects of molecules. As depicted in Figure 10, the atoms of a molecule were conceptualized as nodes within a graph, while the chemical bonds were construed as edges, collectively forming a graph-based representation of the entire molecule. Transformer-based molecular encoding calculates the impact of all atoms on the current atom. This results in even distant and unimportant atoms contributing to the representation of the current atom. As shown in Figure 10B, graph attention networks only consider the influence of directly linked atoms on the current atom, helping the model focus on more important atoms. Through the construction of molecular graphs and the utilization of the GAT model to learn the relationships between atoms, a more comprehensive grasp of the molecular topology could be achieved. During the training process, for each atomic node, GAT dynamically adjusted the weights based on the strengths of connections with neighboring atoms, thereby directing heightened attention toward atoms that bore more relevance. Consequently, the GAT model adeptly accentuated crucial connectivity patterns within the molecular representation, concurrently disregarding less significant elements, and thereby facilitating a more effective expression of the molecular topology.

We commenced by employing one-hot encoding to represent atomic attributes, encompassing atom type, degree, amount of hydrogen, and implicit valence. Subsequently, the interatomic relationships within the molecule were established, employing a two-layer graph attention for the encoding of graph structure. As expressed in Equation (1), the one-hot tensor of each atom was subjected to a linear transformation, projecting it into a higher-dimensional space. Here, *h_j_* signifies the feature representation of atom *j*, W denotes the transformation matrix, and *||* denotes the concatenation of the features of atom *i* and atom *j*. Following the application of the *LeakyReLU* activation function, the resultant *e_i,j_* emerged, representing the weight indicative of the influence of atom *j* on atom *i*. Equation (2) encapsulated the process of aggregating all connected atoms to atom *i* and subsequently normalizing the aggregated values. Incorporating a multi-head attention mechanism, as depicted in Equation (3), the elevated-dimensional feature representation of atom *i* was updated. The Sigmoid function *(σ)* was employed as the activation function, yielding the refined atom feature *h^′^_j_*, which was subject to the dropout function. Following the passage of the molecule through the two layers of graph attention networks, a *ReLU* activation function was applied, succeeded by a global maximum pooling operation. It is pertinent to note that our graph encoder was exclusively operational during the training phase; during inference, its functionality was suspended due to the unavailability of molecular graph structural information.

(1) $$e_{ij}=LeakyReLU(\alpha^{T}[Wh_{i}||Wh_{j}])$$

(2) $$a_{ij}=\frac{exp(e_{ij})}{\sum_{k=1}^{k\in N_{i}}exp(e_{ik})}$$

(3) $$h_{i}^{\prime }=\prod_{k=1}^{K}\sigma \left(\underset{j\in N_{i}}{\sum }\alpha_{ij}^{k}W^{k}{\vec{h}}_{j}\right)$$

### 4.3. GPT encoding of SMILES and Properties

**Figure 11:**
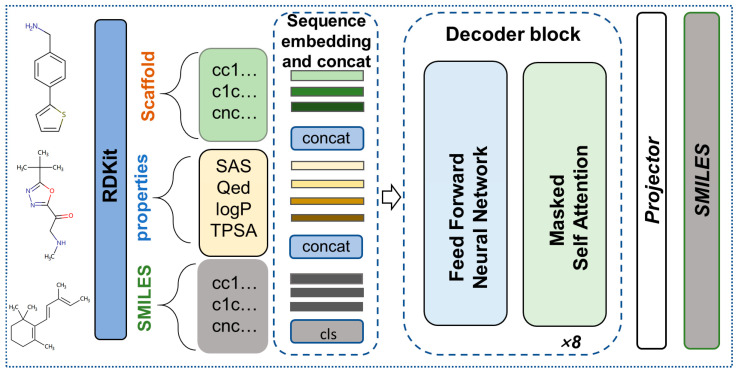
MiniGPT encoding of SMILES; properties and scaffold of molecules.

The molecules were portrayed as sequences in the form of SMILES, serving as the input for the model. Additionally, RDKit [44] was utilized to procure molecular descriptors such as SAS, QED, logP, and TPSA. During the training phase, the molecular properties were concatenated with the respective molecular SMILES, enabling the model to discern the intricate associations existing between molecular properties and SMILES (Figure 11). In the inference stage, predefined molecular properties were specified, facilitating the achievement of conditional molecular generation.

In fact, our model employed a decoder module akin to the Transformer architecture, comprising 8 stacked decoders: a design reminiscent of architectures found within the GPT series. A comparison between the model we utilized and GPT-1 is provided in Table 6. For the sake of simplicity and efficiency, the number of decoder layers in the model as well as the attention header are compressed. An attention mechanism was employed to discern the influence of individual characters within the SMILES, thereby facilitating the feature updates. The calculation methodology for the attention mechanism is outlined in Equation (4), where *Q*, *K*, and *V* represent the Query, Key, and Value vectors, respectively, *T* represents the transpose, and *d_k_* represents the Key vector dimension. After the sequence representation through the class GPT, the representation of the sequence is mapped to the same space as the representation of the graph encoder through a mapper, which is used with a fully connected representation in this study.

(4) $$\mathrm{Attention}(Q,K,V)=\mathrm{softmax}\left(\frac{QK^{T}}{\sqrt{d_{k}}}\right)V$$

### 4.4. Optimum Objectives

In order to fuse the topological information, we employ a GAT in the molecule generation task and subsequently generate molecules with specific properties. As demonstrated in Equation (5), our formulated loss function encompasses two distinct components. The first loss function, denoted as *L_BT_*, encapsulates the disparity between the molecular representation post-traversal through the graph encoder and SMILES sequence encoder. Given that these representations emanate from differing perspectives in molecular characterization, the features resulting from the graph encoder and the sequential encoder should exhibit close proximity. We constrained sequential encoding by employing the graph-encoded representation, thereby enabling the model to indirectly glean topological structural information inherent to the molecule. The second loss function, denoted as *L_ground_*, encapsulates the divergence between the molecular predictions yielded by the model and the actual molecular structures. As depicted in Equation (6), the computation approach for measuring the gap between the graph structure encoding and the sequence encoding adopts the Barlow Twins loss function [45]. Here, *λ* serves as a hyperparameter, set to 0.005, and *C_ij_* represents the correlation coefficient between the graph structure encoding and the sequence encoding, as calculated in Equation (7). Within these equations, *b* denotes a batch index, while *i* and *j*, respectively, index the graph encoder and sequence encoder. *A* and *B* correspond to the graph encoder and sequence encoder, respectively.

(5) $$L=L_{ℬT}+L_{groud}$$

(6) $$L_{ℬT}=\sum_{i}{\left(1-C_{ii}\right)}^{2}+\lambda \;\sum_{i}\sum_{j\neq i}C_{ij}{}^{2}$$

(7) $$C_{ij}=\frac{\sum_{b}z_{b,i}^{A}z_{b,j}^{B}}{\sqrt{\sum_{b}{\left(z_{b,i}^{A}\right)}^{2}\sqrt{\sum_{b}{\left(z_{b,j}^{B}\right)}^{2}}}}$$

### 4.5. Dataset

We tested GraphGPT for the molecular generation task using two datasets: the GuacaMol dataset and the MOSES dataset. The GuacaMol dataset comprises a subset of 1.6 million molecules from the ChEMBL 24 database [46]. The molecular properties, such as molecular weight, LogP, and the number of rotatable bonds, exhibit heterogeneous distributions within this dataset. The MOSES dataset, containing 1.9 million lead-like compounds derived from the ZINC database [47], was created to represent molecule-like lead compounds. As a result, the molecular distribution in the MOSES dataset adheres more closely to desirable drug-like properties. Notably, the molecular properties within the MOSES dataset align more closely with those of actual drugs, featuring properties, such as logP lower than 7 and greater than 3.5. Given the wider range of molecular property distribution within the GuacaMol dataset, it was utilized for testing the generation of molecules with specified properties. In contrast, the MOSES dataset, closely mimicking attributes of real-world drugs, was employed to test the generation of molecules with designated scaffolds and properties. In both test scenarios, 10,000 molecules were generated using the model for evaluation. We employed the RDKit to calculate molecular properties and extract Bemis–Murcko scaffolds and four properties of the molecule, including Synthetic Accessibility Score, Quantitative Estimation of Drug-likeness, lipophilicity, and Topological Polar Surface Area.

### 4.6. Metrics

We used six metrics to assess the effectiveness as well as the diversity of generated molecules by the model, and here is what the metrics mean:

- Valid: Valid pertains to the valid portions within the generated molecules based on SMILES syntax and atomic valency rules. We consider a molecule valid when the generated SMILES can be analyzed using an RDKit. A high valid score indicates that the model has learned the accurate representation of molecules and their chemical properties.
- Unique: Unique specifies that it is a case of duplicates in the generated molecule. If the newly generated molecule has not been generated before, then it is considered ideal. A lower uniqueness score suggests that the model is generating repetitive or redundant molecules.
- Novelty: Novelty refers to the segments present in the generated valid and unique molecules that are absent in the training dataset. This metric is employed to determine whether the model is overfitting, signifying that it has memorized the training data without generalizing to unseen molecules.
- Internal Diversity (IntDivp): Internal Diversity evaluates the similarity between generated molecules. As shown in Equation (8), *s*1 and *s*2 denote two molecules, and *T* represents Tanimoto similarity [48]. This entails similarity comparisons between all pairs of molecules within the generated set (*S*). The parameter *p* can be either 1 or 2.
  (8) $$IntDiv_{p}(S)=1-\sqrt[{p}]{\frac{1}{|S{|}^{2}}\sum_{s1,s2\in S}T{\left(s1,s2\right)}^{p}}$$
- Frechet ChemNet Distance (FCD): This metric tests the similarity of the generated molecular data to the training molecular data. As shown in Equation (9), where *μ_G_* is the mean and Σ*_G_* is the covariance of the distribution *G*. In the same way as Bagal et al. [36] for the Guacamol data set, the final value is −0.2 power of *FCD*.
  (9) $$FCD(G,D)=||\mu_{G}-\mu_{D}|{|}^{2}+Tr(\Sigma_{G}+\Sigma_{D}-2{\left(\Sigma_{G}\Sigma_{D}\right)}^{1/2})$$
- KL Divergence: KL Divergence was computed using a plethora of physicochemical descriptors for both the generated molecules and the training set. Lower values denote a proficient learning of the distribution of these properties by the model. The calculation is shown in Equations (10) and (11). Here, *k* represents the *k*th properties.
  (10) $$D_{KL}(P,Q)=\sum_{i}P(i)\log\frac{P(i)}{Q(i)}$$
  (11) $$S=\frac{1}{k}\sum_{i}^{k}\exp(-D_{\mathrm{KL},i})$$

### 4.7. Baselines

We juxtaposed our model against seven distinct baseline models, encompassing CharRNN [49], VAE [50], AAE [51], JTN-VAE [52], LatentGAN [53], ORGAN [54], and MolGPT.

## 5. Conclusions

The primary contribution of this work lies in the successful integration of graph structures into an NLP-inspired sequence-to-sequence framework. This novel approach not only harnesses the power of language modeling but also seamlessly captures and exploits the topological features of molecules. Our model demonstrates exceptional performance in generating molecules with higher validity, uniqueness, and diversity. Unconditional molecule generation, property molecule generation, and scaffold-based molecule generation experiments all demonstrate the performance of our model. Through extensive experimentation and evaluation of established datasets, we showcase GraphGPT making significant strides in achieving more advanced structure-aware molecular generation techniques. Our method facilitates the construction of large-scale molecular screening libraries and the generation of lead compounds with specified properties, thereby propelling advancements in AI-driven drug discovery and related fields.

## Figures and Tables

**Table 1:** Single property molecule generation, tested with the GuacaMol dataset.

	Model	Valid ↑	Unique ↑	Novelty ↑	SD ↓	MAD ↓
logP	MolGPT	**0.971**	0.998	1	0.31	0.23
	ours	0.969	0.998	1	**0.29**	**0.22**
TPSA	MolGPT	**0.972**	0.996	1	4.66	3.52
	ours	0.971	**0.997**	1	**4.21**	**3.31**
QED	MolGPT	**0.977**	0.995	1	0.20	0.13
	ours	0.968	**0.999**	1	**0.07**	**0.05**
SAS	MolGPT	0.975	**0.998**	1	0.20	**0.13**
	ours	**0.977**	0.996	1	**0.19**	0.14

**Table 2:** Multi property molecule generation, tested with the GuacaMol dataset.

	Model	Valid ↑	Unique ↑	Novelty ↑	SD/MAD ↓		
					logP	SAS	TPSA
logP + SAS	MolGPT	**0.972**	**0.992**	1	0.340/**0.250**	0.210/**0.140**	
	ours	0.971	0.991	1	**0.331**/0.252	**0.208/**0.151	
SAS + TPSA	MolGPT	**0.971**	**0.988**	1		0.220/**0.150**	4.940/3.760
	ours	0.970	**0.988**	1		**0.217/**0.158	**4.705/3.647**
logP + TPSA	MolGPT	**0.965**	0.994	1	0.320/**0.240**		4.770/3.710
	ours	0.961	**0.995**	1	**0.314/0.240**		**4.575/3.607**
logP + TPSA + SAS	MolGPT	**0.973**	**0.969**	1	0.350/0.270	0.260/**0.180**	4.800/3.790
	ours	0.966	0.964	1	**0.335/0.259**	**0.247/**0.183	**4.461/3.532**

**Table 3:** Sampling of 10,000 molecules with different metrics for unconditional molecule generation based on a model trained on the GuacaMol dataset.

	Valid ↑	Unique ↑	Novelty ↑	FCD ↑	KL Divergence ↑
SMILES LSTM	0.959	**1**	0.912	0.913	0.991
AAE	0.822	**1**	0.998	0.529	0.886
Organ	0.379	0.841	0.687	0	0.267
VAE	0.87	0.999	0.974	0.863	0.982
MolGPT	**0.981**	0.998	**1**	0.907	**0.992**
ours	0.975	0.999	**1**	**1.009**	**0.992**

**Table 4:** Sampling of 10,000 molecules with different metrics for unconditional molecule generation based on a model trained on the Moses dataset.

	Valid ↑	Unique ↑	Novelty ↑	IntDiv1 ↓	IntDiv2 ↓
charRNN	0.975	0.999	0.842	0.856	0.850
VAE	0.977	0.998	0.695	0.856	0.850
AAE	0.937	0.997	0.793	0.856	0.850
LatentGAN	0.897	0.997	**0.949**	0.857	0.850
JT-VAE	**1**	0.999	0.914	0.855	0.849
MolGPT	0.994	**1**	0.797	0.857	0.851
ours	0.995	0.999	0.787	**0.851**	**0.845**

**Table 5:** Impact of different number of decoder layers and graph encoders on the model.

	Graph Encoder	Decoder Layer	Valid ↑	Unique ↑	Novelty ↑	SD ↓	MAD ↓
miniGPT_a	×	4	0.946	0.999	1	5.017	3.806
miniGPT_b	×	8	0.972	0.997	1	4.474	3.485
miniGPT_c	×	12	0.977	0.996	1	4.240	3.238
GraphGPT	√	8	0.971	0.997	1	4.208	3.307

**Table 6:** Comparison of GPT-1 and GraphGPT.

	GPT-1	GraphGPT
Decoder layer	12	8
Attention header	12	8
Dimensions of vocab	768	256
Sequence length	512	100
Parameter	117 million	7.07 million

## Data Availability

The Moses and GuacaMol datasets are openly available. Our model is available via: https://github.com/luhao27/GraphGPT. accessed on 22 November 2023.
